# Turkish Version of the New Instrument for Orthorexia Nervosa—Test of Orthorexia Nervosa (TON-17): A Validity and Reliability Study

**DOI:** 10.3390/nu15143178

**Published:** 2023-07-18

**Authors:** Emine Yassıbaş, Feride Ayyıldız

**Affiliations:** Department of Nutrition and Dietetics, Faculty of Health Sciences, Gazi University, 06490 Ankara, Turkey; feridecelebi@gazi.edu.tr

**Keywords:** orthorexia nervosa, TON-17, adaptation, eating attitudes, obsessive beliefs

## Abstract

This study aimed to adapt the Test of Orthorexia Nervosa- (TON-17) into Turkish and verify its validity and reliability. The study included 539 adults with a mean age of 30.2 ± 12.26 years. A reliability analysis was performed, along with a confirmatory factor analysis to test its construct validity. The time-invariance of the scale was examined by test–retest analysis, and its convergent validity was evaluated by a correlation analysis conducted to test the relationships between the scale and Eating Attitudes Test-26 and Obsessive Beliefs Questionnaire-9. Analyses were conducted using SPSS Version 28 and the AMOS-24 software. The Cronbach’s α internal consistency coefficient of the total scale was found to be 0.82, suggesting a strong internal consistency. The Cronbach’s α values of its factors were 0.68 for the “control of food quality”, 0.64 for the “fixation on health and a healthy diet”, and 0.73 for the “disorder symptoms”. In addition, the test–retest reliability was found to be 0.87 for the total scale, suggesting excellent reliability. Most of the fit indices (CMIN/df, RMSEA, AGFI, NFI, and TLI) of the scale were acceptable, and the GFI indicated a good model fit. This study shows that the Turkish version of TON-17, which is a new tool with a three-factor structure to evaluate orthorexia, is a valid and reliable scale.

## 1. Introduction

Orthorexia nervosa (ON) was first defined by Bratman in 1997 as “unhealthy obsession with healthy eating” [1]. In the current literature, ON is defined as “pathological obsession, fixation or preoccupation with healthy foods” [2]. ON is characterized by excessive concentration on food quality, food preparation, and nutrition [3]. ON has several symptoms, including avoidance of food additives such as preservatives, colors, flavorings, pesticides, excessive fat, sugar, and salt, or of genetically modified organisms [3,4]. In addition, individuals with ON may be obsessed with cooking methods and tools in the food preparation process, and they may feel guilt and fear when they go beyond their norms in this process [1,5]. Thoughts on healthy nutrition, food preparation, and labeling of foods can negatively affect both quality of life and social life in individuals with ON [6].

ON is not yet considered to be a psychiatric eating disorder, and it is not included in the 11th revision of the International Classification of Diseases (ICD-11) [7] or in the Diagnostic and Statistical Manual of Mental Disorders, Fifth Edition (DSM-5) [8]. While there is no consensus on the definition of ON so far, a multidisciplinary cohort of experts (47 eating disorder researchers and multidisciplinary treatment specialists) published a consensus document on the definition and diagnostic criteria for ON in 2022 [9]. In this consensus document, ON was characterized by “a strong preoccupation with one’s eating behavior and with self-imposed rigid and inflexible rules which are strictly controlled and include spending an excessive amount of time for planning, obtaining, preparing and/or eating one’s food” [9]. Although a consensus has been published on 27 criteria in the definition and diagnosis of ON, it seems that there is no gold-standard scale used in diagnosis.

A recent systematic review reported a total of 10 different scales to evaluate ON [2], including the Body Image Screening Questionnaire (BISQ) [10], the Burda Orthorexia Risk Assessment (B-ORA) [11], Bratman’s test (Orthorexia self-test—BOT) [12], the Düsseldorf Orthorexia Scale (DOS) [13], the Eating Habits Questionnaire (EHQ) [14], the Revised Eating Habits Questionnaire (EHQ-R) [15], the Orthorexia Nervosa Scale (ONS) [16], the ORTO-15 Questionnaire [17], the Scale to Measure Orthorexia in Puerto Rican Men and Women [18], and the Teruel Orthorexia Scale (TOS) [19]. In addition, recent studies with the Barcelona Orthorexia Scale (BOS) [20] and the Orthorexia Nervosa Inventory (ONI) [21] are also included in the literature. Among these scales, the BISQ, ORTO-15, BOT, and ONS have low internal consistency, and most of them (e.g., BISQ, BOT, and ONS) do not have test–retest reliability; some (DOS, TOS) have challenging preliminary diagnostic criteria, and others (EHQ-R, ONS, B-ORA) need further evaluation [2]. Meule et al. [22] reported good internal reliability for BOT, EHQ, and DOS and unacceptable internal reliability for ORTO-15. In addition, the authors stated that BOT, EHQ, and DOS were highly correlated with one another and moderately correlated with ORTO-15 [22].

Valente et al. [23] conducted a critical literature review about existing scales for ON and suggested that ON should be reconceptualized, relevant qualitative data collection techniques should be determined to gain insight into its diagnosis, and a new scale should be developed for its assessment. In line with the recommendations of Valente et al. [23], Rogowska et al. [24] developed a new scale for ON in 2021. The Test of Orthorexia Nervosa-40 (TON-40), which initially included 40 items, was reduced to 17 items (Test of Orthorexia Nervosa-17 (TON-17)) as a result of structural analysis. TON-17 and its factors have good psychometric properties, stability, reliability, and construct validity. It consists of three factors: control of food quality, fixation on health and healthy diet, and disorder symptoms [24].

In Turkey, the ORTO-11 scale—a short version of ORTO-15—is used frequently in studies to determine ON tendencies [25]. However, there are contradictions in the results of these studies in terms of validity and reliability, with a suggestion of high ON frequencies. In addition, the ONI has recently been adapted into Turkish by Kaya et al. [26], and it was stated to be a valid and reliable scale. The ONI contains 24 questions and has no cutoff point [26]. Based on this, it was thought that TON-17 could be a useful tool for clinicians in academic research and practice, especially since it is a short-form scale and has a cutoff point. This study will contribute to the literature by evaluating the validity and reliability of the Turkish version of TON-17, which was developed in line with the current literature.

## 2. Materials and Methods

### 2.1. Participants

A questionnaire created using Google Forms was used as a data collection tool and sent to individuals via e-mail or WhatsApp, and the individuals were selected by snowball sampling. Those who agreed to participate in the study completed the questionnaire online after giving their consent. All participants were Turkish citizens who could speak Turkish and lived in Turkey. This study was conducted with healthy adults, and individuals diagnosed with eating disorders or psychiatric diseases were not included in the study, as this may have had a confounding effect. Validity and reliability studies have reported that the sample size should be 5–10 times the number of scale items [27]. The scale used in this study consists of 17 items; therefore, we aimed to reach at least 85–170 individuals for conducting the study. A total of 539 adults aged between 18 and 65 years, including 131 males (24.3%) and 408 females (75.7%), participated in this study.

### 2.2. Design and Procedure

**Figure 1:**
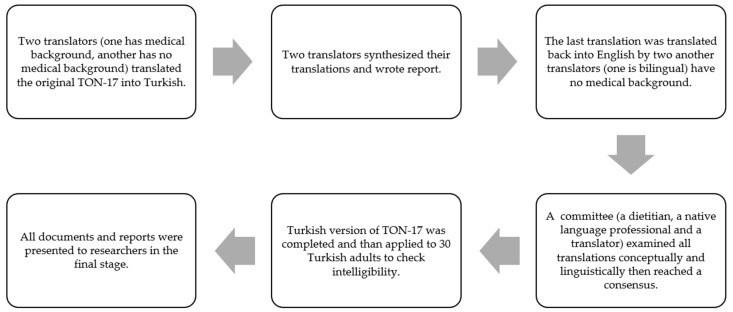
Process of cross-cultural adaptation of TON-17.

To adapt TON-17 into Turkish, permission was obtained from Aleksandra Rogowska [24] (one of the researchers who developed the scale) via e-mail. TON-17 was translated according to the guide created by Beaton et al. [28]. The process of cross-cultural adaptation of TON-17 was as shown in Figure 1.

The reliability of the questionnaire was tested with test–retest and internal consistency analyses. To examine its test–retest reliability, the final version of the Turkish TON-17 was administered to 30 adult volunteers twice, with an interval of 15 days.

The questionnaire, which was prepared with the final version of the Turkish TON-17 (see the Supplementary Materials), was applied online. The questionnaire collected information about the participants’ sociodemographic characteristics (such as age, gender, and educational status). Their body weight (kg) and height (cm) were self-reported. To evaluate the convergent validity of TON-17, the Eating Attitudes Test Short-Form (EAT-26) [29,30] and the Obsessive Beliefs Questionnaire Short-Form-9 (OBQ-9) [31,32] were used. The eating behaviors of the participants were evaluated with EAT-26, and obsessive disorders were evaluated with OBQ-9.

### 2.3. Measurements

Test of Orthorexia Nervosa (TON-17)

The Test of Orthorexia Nervosa (TON-17) is a self-report scale developed by Rogowska et al. [24] in 2021 to evaluate ON. Based on the scientific literature review and interviews with people at risk of orthorexia, it was first developed as 40 items (TON-40) to test ON, and the number of items was reduced to 17 as a result of structural analysis. TON-17 is considered to be a useful tool to assess the risk of ON. It has three sub-factors (control of food quality (items 1, 4, 7, 10, 13, and 16), fixation on health and healthy diet (items 2, 5, 8, 11, and 14), and disorder symptoms (items 3, 6, 9, 12, 15, and 17)) and a general factor (total of 3 sub-factors). Each item is rated on a five-point Likert-like scale, indicating the degree of compliance with the sentence (1 = strongly disagree, 2 = disagree, 3 = undecided, 4 = agree, 5 = strongly agree). The scale has no reverse scoring. A higher score is associated with a greater risk of ON. The cutoff value for ON is a general factor score equal to or above 61. The internal consistency (Cronbach’s α) was 0.79 for the general factor, 0.80 for the 1st factor (F1) (control of food quality), 0.81 for the 2nd factor (F2) (fixation on health and a healthy diet), and 0.74 for the 3rd factor (F3) (disorder symptoms).

Eating Attitudes Test Short-Form (EAT-26)

This is a 26-question version of the Eating Attitudes Test-40 developed by Garner and Garfinkel [33] in 1979, which was revised and shortened by Garner et al. [30]. The scale was adapted into Turkish by Ergüney-Okumuş and Sertel-Berk [29]. In addition to its ease of use and scoring, this scale provides advantages in terms of psychometric properties, economy, and practicality. The Cronbach’s α internal consistency coefficient of the scale was found to be 0.84 in the Turkish adaptation study [29]. In this study, the Cronbach’s α of the scale was determined to be 0.80.

EAT-26 is used to detect and define eating disorder behaviors in healthy individuals and to diagnose anorexia nervosa and bulimia nervosa, which are also called eating disorders. All scale items except for the 26th item are scored as “3 = Always, 2 = Very often, 1 = Often, 0 = Sometimes, 0 = Rarely, 0 = Never”. However, the 26th item is scored in reverse as “1 = Sometimes, 2 = Rarely, and 3 = Never”, where other options are worth 0 points. A scale score of 20 or above indicates deterioration in eating behavior, and a higher score is associated with more evident eating disorder [29].

Obsessive Beliefs Questionnaire Short-Form-9 (OBQ-9)

The Obsessive Beliefs Questionnaire (OBQ), which was developed by an international working group to evaluate cognitive biases specific to obsessive compulsive disorder and consisted of 87 items in its initial version, was later used as OBQ-44 with 44 items [34,35]. Gagné et al. [31] developed a 9-item short version in 2018. The Turkish validity and reliability study of the Obsessive Beliefs Questionnaire Short-Form was conducted in 2019 by Yorulmaz et al. [32]. The 9-item short form of the OBQ, which has 3 subscales (perfectionism and intolerance to uncertainty (Cronbach’s α = 0.75), responsibility and threat perception (Cronbach’s α = 0.74), and importance and control over thoughts (Cronbach’s α = 0.70)), was used in this study. Both the total score and the 3 sub-dimension scores of the scale can be used separately [32]. In this study, the Cronbach’s α was found to be 0.76 for the total OBQ-9.

Body Mass Index (BMI)

The participants’ body weight (kg) and height (cm) values were self-reported. BMI was calculated by dividing the body weight in meters by the square of the height in meters (kg/m^2^), considering the WHO’s classification (BMI: <18.5 kg/m^2^ = underweight, BMI: 18.5–24.9 kg/m^2^ = normal, BMI: 25.0–29.9 kg/m^2^ = overweight and BMI: >30.0 kg/m^2^ = obese) [36].

### 2.4. Statistical Analysis

The data were analyzed using IBM SPSS version 28 and the AMOS-24 software. Descriptive statistics were used to analyze the general characteristics of the sample. Skewness and kurtosis tests were used to determine whether the data were normally distributed. Cronbach’s alpha and McDonald’s omega coefficients were used to determine the reliability and internal consistency of the Turkish version of TON-17, where values above 0.60 and 0.70 were considered to show acceptable and good fits, respectively [27]. Test–retest reliability was evaluated with the correlation coefficient calculated using the data obtained from a subsample of 30 participants who filled out TON-17 twice. A confirmatory factor analysis (CFA) was performed using the AMOS-24 software to determine the validity of the scale and to analyze the compatibility of its subscales. Maximum likelihood was used to observe parameter estimation for CFA. The multiple fit indices, including CMIN/df (chi-squared goodness of fit), RMSEA (root-mean-square error of approximation), GFI (goodness-of-fit index), AGFI (adjusted goodness-of-fit index), NFI (normed fit index), and TLI (Tucker–Lewis index), were evaluated within the scope of the CFA [37]. The convergent validity was examined using Pearson’s or Spearman’s correlation coefficients between TON-17 and other scales, depending on whether the data were normally distributed or not. The significance level was accepted as 0.05 in all statistical analyses.

## 3. Results

This study evaluated the validity and reliability of the Turkish version of TON-17, using data obtained from a total of 539 adults, including 131 males (24.3%) and 408 females (75.7%). The general characteristics of the participants are presented in Table 1. Their mean age was 30.2 ± 12.26 years. Of the participants, 82.2% had received a university education, 69.2% were single, 76.1% did not smoke, 75.1% did not use alcohol, 57.0% had normal body weight, 27.3% were overweight, and 7.6% were obese. The mean BMI was 26.2 ± 4.16 kg/m^2^ for males and 22.9 ± 3.94 kg/m^2^ for females.

Like the original TON-17, a hierarchical bi-factor model was found to be suitable for the Turkish version, with three lower-order factors (control of food quality, fixation on health and healthy diet, and disorder symptoms) and one general higher-order factor (orthorexia). The Cronbach’s α internal consistency coefficient was found to be 0.82 and McDonald’s omega was found to be 0.86 for the general factor, suggesting a strong internal consistency. The Cronbach’s α was found to be 0.68 for factor 1, 0.64 for factor 2, and 0.72 for factor 3; similarly, McDonald’s omega was found to be 0.68 for factor 1, 0.63 for factor 2, and 0.70 for factor 3. In the analysis performed to evaluate the test–retest reliability of the Turkish version of TON-17, a significant positive correlation was found for both the general factor and the sub-factors (*p* < 0.005) (Table 2).

The mean scores of the items and factors of TON-17 are presented in Table 3. The mean score for the first factor was 17.9 ± 3.92, that for the second factor was 17.0 ± 2.92, that for the third factor was 13.3 ± 3.78, and that for the general factor was 48.1 ± 8.55.

**Figure 2:**
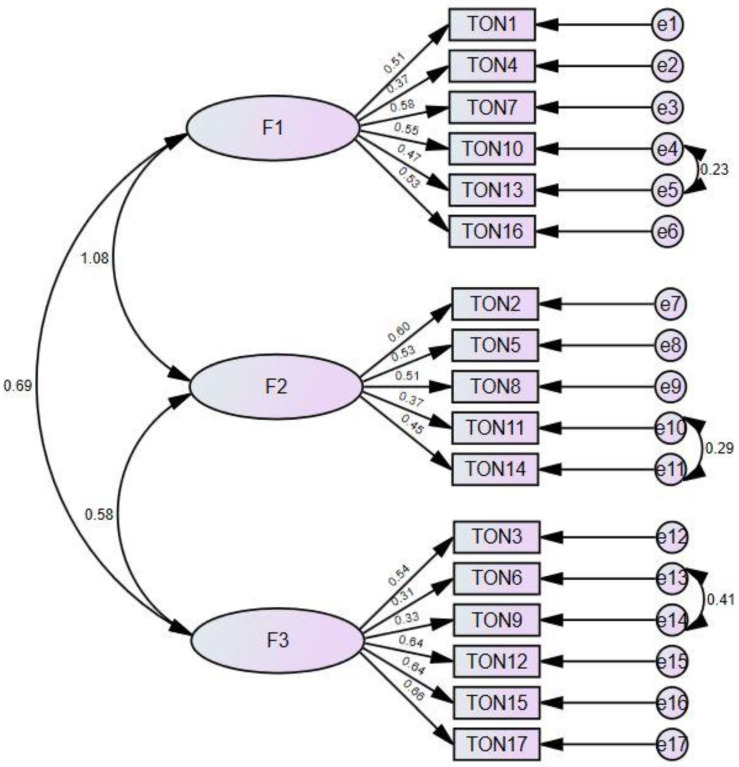
Confirmatory factor analysis of TON-17 and the three-factor model path diagram. F1: control of food quality, F2: fixation on health and healthy diet, F3: disorder symptoms.

Table 4 presents the fit indices of the model, and Figure 2 shows the three-factor model fit diagram. Among the goodness-of-fit indices, CMIN/df (χ^2^/df) was found to be 3.81, RMSEA was 0.07, GFI was 0.90, AGFI was 0.87, NFI was 0.80, and TLI was 0.81 (Table 4). On the basis of CFA, the standard factor loading of the scale varied between 0.37 and 0.58 for factor 1, between 0.37 and 0.60 for factor 2, and between 0.31 and 0.66 for factor 3. Accordingly, the factor loading of TON-17, which consists of 3 factors and 17 items, was found to be at an acceptable level (Figure 2).

Table 5 presents the correlations of TON-17’s general factor and sub-factors with the EAT-26 and OBQ-9 scales. The general factor of TON-17 had a significant low-level positive correlation with EAT-26 (r:0.29) and OBQ-9 (r:0.35) (*p* < 0.001).

The mean EAT-26 score was 8.5 ± 7.35, and the mean OBQ-9 score was 38.8 ± 8.73. In addition, 7.4% of the individuals were found to have ON, considering the cutoff score for TON-17 as ≥61, and 8.4% of the individuals were found to have eating disorders, considering the cutoff score for EAT-26 as ≥20. In addition, the EAT-26 and OBQ-9 scores of individuals with and without ON were evaluated, as shown in Table 6. The EAT-26 total score, OBQ-9 total score, and OBQ-9 subscale scores were found to be significantly higher in adults with ON (*p* < 0.001). The observed effect sizes were medium (>0.50) or large (>0.80).

## 4. Discussion

ON is characterized by consuming healthy foods and focusing on their content and quality [22], and it can be briefly defined as an obsession with healthy eating [38]. This behavior may include an unbalanced diet due to beliefs about the “purity” of food, strict avoidance of unhealthy foods, feelings of guilt and anxiety after eating violations, or intolerance to other people’s food beliefs [22,39]. Extreme orthorexic behaviors may cause physical health to deteriorate due to malnutrition and cause social and academic failures due to obsessive thoughts and behaviors focused on beliefs about healthy eating [39]. Donini et al. [9] also stated in the consensus report that ON-related behaviors negatively affect health status and quality of life. Therefore, the diagnosis and treatment of ON may be important in improving health and quality of life.

In the literature, different scales (BOT, ORTO-15, EHQ, DOS, TOS, BOS, and ONI) have been used to evaluate ON [3], and their positive and negative aspects have been discussed [3,4,38]. A decrease in the total score on ORTO-15—one of the most frequently used scales in the literature—indicates higher orthorexic behavior [38]. While the individuals with the lowest scores are defined as having ON, some of them have been observed to have healthy eating behaviors and normal personalities, and this has not been discussed by researchers [3,38]. In the following years, several versions of ORTO-15 with different item numbers, factor numbers, and cutoff scores were developed [25,40,41,42]. Therefore, the usability of ORTO-15 has been questioned by many studies due to the high frequency of ON in the groups studied, the lack of information about how the items are created, and the lack of a clear, validated tool [3,4,38]. The use of ORTO-15 is considered dubious due to these limitations, and its use is not recommended, although it is popular.

In a systematic review to examine the psychometric properties of scales assessing ON, it was stated that there is no gold standard for assessing ON [2]. Thus, future studies will be important for establishing a gold standard for determining the risk of ON [24]. In this regard, the validity and reliability studies of TON-17 in different societies will contribute to the literature. The TON-17 scale was developed based on qualitative methods (e.g., interviews) and a reconceptualization of ON using cutting-edge technology. In addition, statistical techniques such as EFA, CFA, and CCA supported the structural construction of TON-17. TON-17 consists of three sub-factors, and this structure for ON has been used previously in DOS, EHQ, and ORTO-15 [2]. In TON-17, sub-factors F1 and F2 are both associated with healthy orthorexia, while F3 is associated with unhealthy orthorexia [24]. More recently, it has been proposed to distinguish between ON, as defined here, and “healthy orthorexia”, which refers to a non-pathological interest in healthy eating and food culture [43,44,45].

This study was conducted to determine the validity and reliability of the Turkish version of TON-17 [24]. Similar to the original TON-17 (Cronbach’s α 0.79 for the general factor), the reliability analysis of the Turkish version of TON-17 suggested good internal consistency (Cronbach’s α 0.82) for the general factor. The Cronbach’s α coefficients for the three sub-factors of the scale were lower than those in the original study (0.80, 0.81, and 0.74 for F1, F2, and F3, respectively), but still at an acceptable level (0.68, 0.64, and 0.72 for F1, F2, and F3, respectively). The Cronbach’s α coefficient of the ORTO-15 scale, which is the most frequently used for evaluating orthorexia in Turkey, is 0.62, and the internal consistency of TON-17 was found to be higher than that of ORTO-15 [25]. On the other hand, the internal consistency of the Turkish ONI (Cronbach’s α 0.91) [26] was higher than that of TON-17 (Cronbach’s α 0.82). However, the lack of test–retest reliability of the Turkish ONI may be a disadvantage, and it also has no cutoff point. In this study, the test–retest reliability was found to be 0.87 for the general factor, suggesting excellent reliability for the Turkish TON-17. Most of the fit indices (CMIN/df, RMSEA, AGFI, NFI, and TLI) obtained by CFA were found to be at acceptable or good (GFI) levels. Accordingly, TON-17 can be considered to be a valid and reliable tool to determine the risk of ON in Turkish adults, and it is associated with both eating disorders and obsessive behavior.

Clinical observation and existing research have indicated that ON may share important traits with both eating disorders and obsessive–compulsive disorder (OCD) [46]. In a recent meta-analysis examining the associations between ON, eating disorders, and obsessive–compulsive symptoms, ON symptoms were shown to be more associated with eating disorders (r:0.36, *p*: < 0.01) than with OCD (r:0.21, *p* < 0.01) [47]. However, in this study, parallel to the original study [24], it was found that the TON-17 general score was more correlated with general OCD scores (r:0.35, *p* < 0.01) compared to the EAT-26 scores (r:0.29, *p* < 0.01) (Table 5). The fact that the relationships were not strong is a recurring theme throughout both this study and the meta-analysis. On the other hand, it should be considered crucial that the EAT-26 total score, OBQ-9 total score, and OBQ-9 subscale scores were found to be significantly higher in adults with ON in this study, since studies using different ON scales have shown similar results in the literature [48,49,50].

Niedzielski et al. [3] have reported that there are no reliable data on the prevalence of ON, and that there are differences in the frequency of ON depending on the diverse cutoff scores and tools used in the existing studies. Due to the lack of standardized diagnostic tools, there is a large variability in the prevalence of ON in the literature, ranging from 0% to 97% [51,52]. The prevalence of ON varies by country of study, participant profile, and the tool used to assess ON in the study. While the prevalence of ON was 6.9% [53] in one study using ORTO-15, it was 75.2% in another study [54]. It has been reported that the use of ORTO-15 to diagnose ON should be questioned due to the high rate of false positive results [3]. The prevalence of ON was found to be 5.5% in the study of Rogowska et al. [24]. Also, the prevalence of ON was found to be 7.5% in the present study. Future studies of TON-17 in diverse cultures will contribute to the literature.

The Turkish validity and reliability of a scale for orthorexia, which is the cutoff point, is one of the strengths of this study. In addition, the retest was performed in the study, and the results were statistically significant, constituting another strength of this study. Although it was conducted with a large sample, this study has some limitations that should be noted. One of the limitations of this study is that the majority of the participants were women. It is thought that this may be related to the fact that women show more interest in studies related to nutrition. In addition, the high level of education of most of the participants makes it difficult to generalize the findings to the adult population.

## 5. Conclusions

In conclusion, this study has shown the validity and reliability of the Turkish version of TON-17—a new tool with a three-factor structure to evaluate orthorexia. This scale will contribute to the literature, allowing future studies to better examine the prevalence, risk, and diagnosis of ON.

## Figures and Tables

**Table 1:** General characteristics of participants (*n* = 539).

Variables	X ± SD or *n* (%)	Min	Max
Age (years)	30.2 ± 12.26	18	64
Gender			
Male	131 (24.3%)		
Female	408 (75.7%)		
BMI (kg/m^2^) *	23.6 ± 4.12	15.06	38.25
Underweight	44 (8.1%)		
Normal weight	307 (57.0%)		
Overweight	147 (27.3%)		
Obesity	41 (7.6%)		
Marital status			
Single	373 (69.2%)		
Married	166 (30.8%)		
Education level			
Elementary school	4 (0.8%)		
High school	23 (4.2%)		
University	443 (82.2%)		
Postgraduate	69 (12.8%)		
Smoking			
Yes	129 (23.9%)		
No	410 (76.1%)		
Drinking alcohol			
Yes	134 (24.9%)		
No	405 (75.1%)		

* BMI: body mass index.

**Table 2:** Reliability analyses for TON-17.

TON-17	Item	Cronbach’s α Internal Consistency Coefficients	McDonald’s Omega	Test–Retest Reliability Coefficients *	
				r	*p*
F1	6	0.68	0.68	0.86	<0.01
F2	5	0.64	0.63	0.54	<0.01
F3	6	0.72	0.70	0.78	<0.01
General factor	17	0.82	0.81	0.86	<0.01

* Pearson’s correlation; F1: control of food quality, F2: fixation on health and healthy diet, F3: disorder symptoms.

**Table 3:** The scores of TON-17’s items.

Factors and Items	Min	Max	X ± SD
Factor 1	6	30	17.9 ± 3.92
1. I am concerned about too much unhealthy food being available.	1	5	3.5 ± 1.10
4. I don’t trust food prepared by another person.	1	5	2.6 ± 0.98
7. Before I eat something, I make sure that the product has the appropriate health food quality certificates.	1	5	2.7 ± 1.04
10. I don’t eat GMO foods.	1	5	3.0 ± 1.05
13. I do not accept pesticide-produced foods in my diet.	1	5	3.4 ± 1.02
16. I often talk about healthy foods to convince others to change their diet.	1	5	2.7 ± 1.11
Factor 2	5	25	17.0 ± 2.92
2. I pay a lot of attention to the ingredients of food I buy.	1	5	3.3 ± 1.00
5. I plan each meal in detail.	1	5	2.3 ± 0.91
8. People who eat junk food are putting their lives at risk.	1	5	3.3 ± 1.09
11. Health is most important to me.	1	5	4.1 ± 0.74
14. Eating healthy food significantly affects my quality of life.	1	5	4.0 ± 0.78
Factor 3	6	30	13.3 ± 3.78
3. My diet makes me feel lonely.	1	5	2.1 ± 0.93
6. Due to the current diet, my health deteriorated.	1	5	2.4 ± 1.04
9. My relatives, doctors or other health care workers were concerned about my health condition and suggested that I change my diet.	1	5	2.1 ± 1.09
12. I pushed my hobbies and interests to the background by engaging in a healthy lifestyle.	1	5	2.2 ± 0.89
15. I prefer to eat a healthy meal alone than to go out with friends or family to eat something out.	1	5	2.4 ± 1.05
17. I prefer to eat a healthy meal alone than to go out with friends or family to eat something out.	1	5	2.2 ± 0.95
General factor	20	74	48.1 ± 8.55

**Table 4:** Multiple fit indices.

Index	Value	Thresholds for Acceptable Fit *	Thresholds for Good Fit *
CMIN/df	3.81	≤5.00	≤3.00
RMSEA	0.07	≤0.08	≤0.05
GFI	0.90	≥0.80	≥0.90
AGFI	0.87	≥0.85	≥0.95
NFI	0.80	≥0.80	≥0.95
TLI	0.81	≥0.80	≥0.95

CMIN/df: chi-squared goodness of fit, RMSEA: root-mean-square error of approximation, GFI: goodness-of-fit index, AGFI: adjusted goodness-of-fit index, NFI: normed fit index, TLI: Tucker–Lewis index. * Values of thresholds for acceptable and good fit [37].

**Table 5:** Correlation of TON-17 with the EAT-26 and OBQ-9 scales.

Scales	TON-17							
	F1		F2		F3		FG	
	r	*p*	r	*p*	r	*p*	r	*p*
EAT-26 *	0.19	<0.01	0.26	<0.01	0.25	<0.01	0.29	<0.01
OBQ-9								
F1 (perfectionism and intolerance for uncertainty)	0.17	<0.01	0.15	<0.01	0.22	<0.01	0.23	<0.01
F2 (responsibility and threat overestimation)	0.26	<0.01	0.22	<0.01	0.33	<0.01	0.34	<0.01
F3 (importance of and control over thoughts)	0.12	<0.01	0.11	<0.01	0.32	<0.01	0.24	<0.01
Total score	0.23	<0.01	0.20	<0.01	0.37	<0.01	0.35	<0.01
TON-17								
F1 (control of food quality)			0.68	<0.01	0.41	<0.01	0.87	<0.01
F2 (fixation on health and healthy diet)					0.28	<0.01	0.78	<0.01
F3 (disorder symptoms)							0.73	<0.01
General factor								

* Spearman’s correlation (others show Pearson’s correlation). EAT-26: Eating Attitudes Test-26; OBQ-9: Obsessive Beliefs Questionnaire Short-Form-9; TON-17: Test of Orthorexia Nervosa-17.

**Table 6:** Evaluation of the EAT-26 and OBQ-9 scores according to the TON-17 classification.

	Individuals with ON (n:40)	Individuals without ON (n:499)		
	Mean ± SD	Mean ± SD	*p* *	Effect Size **
EAT-26 scores	13.8 ± 11.03	8.1 ± 6.81	0.002	0.79
OBQ-9 scores				
Total	46.3 ± 7.85	38.2 ± 8.52	<0.01	0.95
F1	16.4 ± 3.00	13.9 ± 3.54	<0.01	0.72
F2	16.2 ± 3.04	13.4 ± 3.42	<0.01	0.81
F3	13.8 ± 4.00	10.9 ± 4.05	<0.01	0.69

* *t*-test, ** Hedges’ g, EAT-26: Eating Attitudes Test-26; OBQ-9: Obsessive Beliefs Questionnaire Short-Form-9; ON: orthorexia nervosa.

## Data Availability

The datasets used during the present study can be obtained from the corresponding author upon reasonable request.

## Supplementary Materials

The following supporting information can be downloaded at: https://www.mdpi.com/article/10.3390/nu15143178/s1. The final version of the Turkish TON-17.
