# Supplementary material for: Turkish Version of the New Instrument for Orthorexia Nervosa—Test of Orthorexia Nervosa (TON-17): A Validity and Reliability Study
Source: Nutrients. 2023 Jul 18;15(14):3178. doi: 10.3390/nu15143178 (PMC10384101; doi:10.3390/nu15143178)
Supplement: Supplementary file 1 [file nutrients-15-03178-s001.zip › nutrients-2460292-supplementary.pdf]

TEST OF ORTHOREXIA NERVOSA (TON-17)

Rogowska, Kwaśnicka, & Ochnik, 2021

Below are some statements that may describe your condition, feelings and behavior. Please read each statement carefully and then select the answer that best reflects your feelings and behavior. Please circle your answer on the scale that shows the degree of compliance with the statement:

1 = Strongy disagree<br>2 = Rather disagree<br>3 = Undecided<br>4 = Rather agree<br>5 = Strongly agree		Strongy disagree	Rather disagree	Undecided	Rather agree	Strongly agree
1.	I am concerned about too much unhealthy food being available.	1	2	3	4	5
2.	I pay a lot of attention to the ingredients of food I buy	1	2	3	4	5
3.	My diet makes me feel lonely.	1	2	3	4	5
4.	I don't trust food prepared by another person.	1	2	3	4	5
5.	I plan each meal in detail.	1	2	3	4	5
6.	Due to the current diet, my health deteriorated.	1	2	3	4	5
7.	Before I eat something, I make sure that the product has the appropriate health food quality certificates.	1	2	3	4	5
8.	People who eat junk food are putting their lives at risk.	1	2	3	4	5
9.	My relatives, doctors or other health care workers were concerned about my health condition and suggested that I change my diet.	1	2	3	4	5
10.	I don't eat GMO foods.	1	2	3	4	5
11.	Health is most important to me.	1	2	3	4	5
12.	I pushed my hobbies and interests to the background by engaging in a healthy lifestyle.	1	2	3	4	5
13.	I do not accept pesticide-produced foods in my diet.	1	2	3	4	5
14.	Eating healthy food significantly affects my quality of life.	1	2	3	4	5
15.	I prefer to eat a healthy meal alone than to go out with friends or family to eat something out.	1	2	3	4	5
16.	I often talk about healthy foods to convince others to change their diet.	1	2	3	4	5
17.	Food quality thoughts torment me most of the day.	1	2	3	4	5

ORTOREKSİYA NERVOZA TESTİ (TON-17)

Aşağıda durumunuzu, duygularınızı ve davranışlarınızı tanımlayabilecek bazı ifadeler bulunmaktadır. Lütfen her ifadeyi dikkatlice okuyun, duygu ve davranışlarınızı en iyi yansıtan cevabı seçin. Lütfen cevabınız için ölçekteki ifadede uygunluk derecesini gösteren rakamı daire içine alın:

	**6 = Kesinlikle katılmıyorum**<br>**7 = Katılmıyorum**<br>**8 = Kararsızım**<br>**9 = Katılıyorum**<br>**10 = Kesinlikle katılıyorum**	**Kesinlikle katılmıyorum**	**Katılmıyorum**	**Kararsızım**	**Katılıyorum**	**Kesinlikle katılıyorum**
1.	Çok fazla sağlıksız yiyeceğin var olmasından endişelenirim.	1	2	3	4	5
2.	Satın aldığım yiyeceklerin içeriğine çok dikkat ederim.	1	2	3	4	5
3.	Beslenme alışkanlıklarım kendimi yalnız hissetmeme neden olur.	1	2	3	4	5
4.	Başka birinin hazırladığı yemeklere güvenmem.	1	2	3	4	5
5.	Her öğünümü ayrıntılı olarak planlarım.	1	2	3	4	5
6.	Şu anki beslenme alışkanlıklarım nedeniyle sağlığım bozuldu.	1	2	3	4	5
7.	Bir şeyler yemeden önce o ürünün, sağlığa uygun gıda kalite sertifikalarına sahip olduğundan emin olurum.	1	2	3	4	5
8.	Abur cubur yiyen insanlar hayatlarını riske atarlar.	1	2	3	4	5
9.	Akrabalarım, doktorlar veya diğer sağlık çalışanları sağlık durumumdan endişe duydular ve beslenme alışkanlıklarımı değiştirmemi önerdiler.	1	2	3	4	5
10.	GDO (genetiği değiştirilmiş organizmalar) içeren yiyecekleri yemem.	1	2	3	4	5
11.	Sağlık benim için en önemli şeydir.	1	2	3	4	5
12.	Sağlıklı bir yaşam tarzını benimsediğim için hobilerimi ve ilgi alanlarımı geri plana attım.	1	2	3	4	5
13.	Pestisit (tarım ilaçları) içeren besinlerin beslenmemde yer almasını kabul etmem.	1	2	3	4	5
14.	Sağlıklı besinler yemek yaşam kalitemi önemli ölçüde etkiler.	1	2	3	4	5
15.	Arkadaşlarım veya ailemle dışarıda bir şeyler yemektense tek başıma sağlıklı bir öğün tüketmeyi tercih ederim.	1	2	3	4	5
16.	Başkalarını beslenme alışkanlıklarını değiştirmeye ikna etmek için sık sık sağlıklı besinlerden bahsederim.	1	2	3	4	5
17.	Besinlerin kalitesine dair düşüncelerim günün çoğunda bana eziyet eder.	1	2	3	4	5
